# Ten Rules for the Management of Moderate and Severe Traumatic Brain Injury During Pregnancy: An Expert Viewpoint

**DOI:** 10.3389/fneur.2022.911460

**Published:** 2022-06-09

**Authors:** Simone Di Filippo, Daniel Agustin Godoy, Marina Manca, Camilla Paolessi, Federico Bilotta, Ainhoa Meseguer, Paolo Severgnini, Paolo Pelosi, Rafael Badenes, Chiara Robba

**Affiliations:** ^1^Department of Biotechnology and Sciences of Life, Anesthesia and Intensive Care, ASST Sette Laghi, University of Insubria, Varese, Italy; ^2^Neurointensive Care Unit, Sanatorio Pasteur, Catamarca, Argentina; ^3^Intensive Care, Hospital Carlos Malbran, Catamarca, Argentina; ^4^Anesthesia and Intensive Care, Policlinico San Martino Hospital, IRCCS for Oncology and Neuroscience, Genova, Italy; ^5^Department of Anesthesiology, University of Rome “Sapienza”, Rome, Italy; ^6^Department of Obstetrics, Hospital Francesc de Borja, Gandia, Spain; ^7^Department of Anesthesiology and Surgical-Trauma Intensive Care, Hospital Clinic Universitari de València, Universitat de València, Valencia, Spain

**Keywords:** pregnancy, traumatic brain injury, intensive care unit, neurosurgery, intracranial hypertension, outcome

## Abstract

Moderate and severe traumatic brain injury (TBI) are major causes of disability and death. In addition, when TBI occurs during pregnancy, it can lead to miscarriage, premature birth, and maternal/fetal death, engendering clinical and ethical issues. Several recommendations have been proposed for the management of TBI patients; however, none of these have been specifically applied to pregnant women, which often have been excluded from major trials. Therefore, at present, evidence on TBI management in pregnant women is limited and mostly based on clinical experience. The aim of this manuscript is to provide the clinicians with practical suggestions, based on 10 rules, for the management of moderate to severe TBI during pregnancy. In particular, we firstly describe the pathophysiological changes occurring during pregnancy; then we explore the main strategies for the diagnosis of TBI taking in consideration the risks related to mother and fetus, and finally we discuss the most appropriate approaches for the management in this particular condition. Based on the available evidence, we suggest a stepwise approach consisting of different tiers of treatment and we describe the specific risks according to the severity of the neurological and systemic conditions of both fetus and mother in relation to each trimester of pregnancy. The innovative feature of this approach is the fact that it focuses on the vulnerability and specificity of this population, without forgetting the current knowledge on adult non-pregnant patients, which has to be applied to improve the quality of the care process.

## Introduction

Traumatic brain injury (TBI) is a common cause of mortality and morbidity worldwide (1, 2). The overall mortality remains high ranging from 30 to 50% (3), and at least 40% of survivors will experience permanent disability (1).

Several algorithms have been proposed over the last years for the management of patients with moderate to severe TBI, especially regarding the monitoring and management of intracranial pressure (ICP) (4, 5).

However, at present, no suggestions are provided in the specific subpopulation of pregnant women and there is a lack of level I evidence in many areas, as stated by Brain Trauma Foundation Guidelines (5). The algorithms currently used for the management of adult patients with TBI are not totally applicable in pregnant patients, primarily because there is insufficient evidence in this population due to their exclusion from most trials on TBI and also because there are important changes in mother's physiology, which alter cardiorespiratory response, tolerance and metabolism of drugs and therefore make difficult the application of the general treatments principles (6, 7).

Although etiologies and incidence of TBI in pregnant patients are similar to the general population, trauma-related mortality for pregnant patients is higher than in any other groups of patients (8).

Therefore, management of TBI during pregnancy is challenging and there is a need for specific protocols to help clinicians in the management of these patients to potentially avoid premature death and morbidities in this population of young and healthy women.

**Figure 1:**
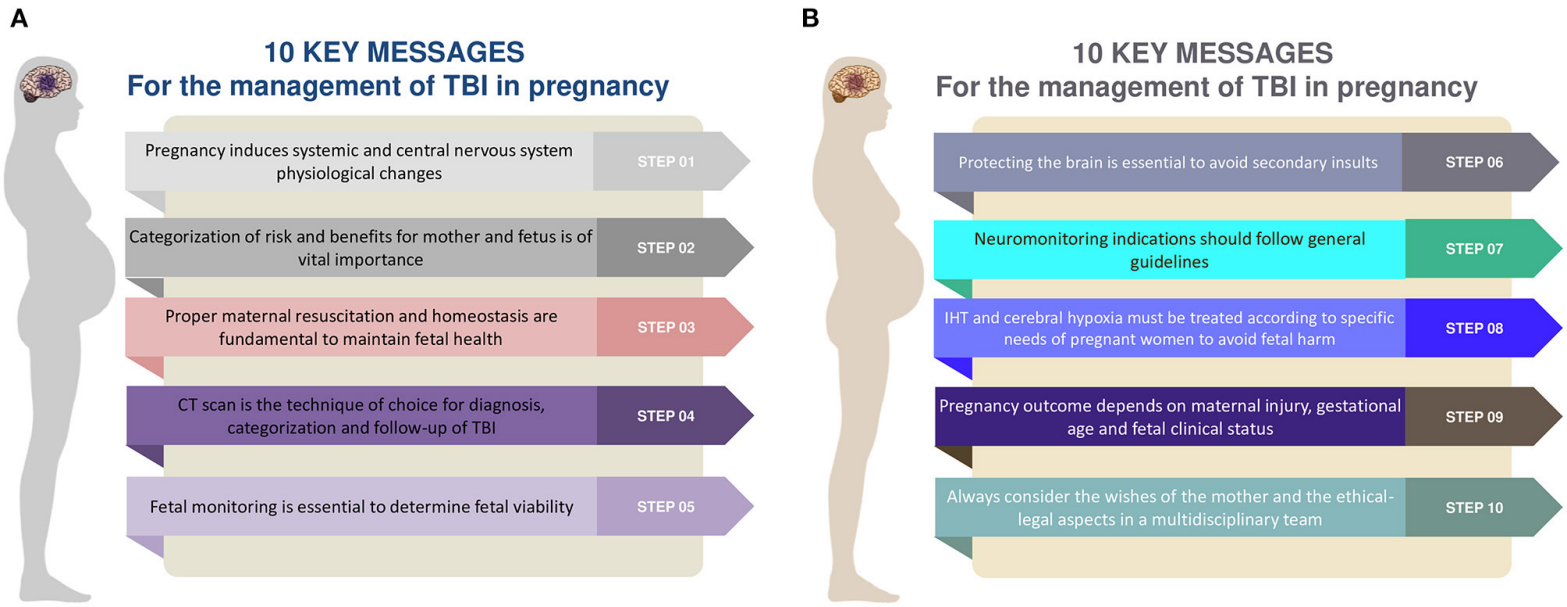
**(A,B)** Ten rules and steps for the management of traumatic brain injury in the pregnant woman. TBI, traumatic brain injury; CT, computed tomography; IHT, intracranial hypertension.

The aim of this review is to provide ten rules and suggestions to apply in the management of moderate to severe TBI in pregnant women, considering pathophysiological changes of pregnancy and differences of specific parameters—such as cerebral perfusion pressure (CPP) and carbon dioxide arterial pressure targets (PaCO_2_) (Figures 1A,B) (9).

We therefore aim to help the clinicians using a combination of evidence, pathophysiological knowledge and common sense in the selection of the best combination of monitoring and medical-surgical intervention, according to the different status of pregnancy, and considering risks and benefits for both mother and fetus.

### Rule 1. Consider Physiological Changes Caused by Pregnancy

Pregnancy prompts a plethora of significant physiological modifications that involve almost every system in the body, aimed to sustain the development of the embryo and fetus (10). The evaluation of a pregnant trauma patient and the interpretation of the diagnostic studies should be therefore performed considering the changes occurring during gestation.

One of the earliest physiological changes that occurs during pregnancy is the substantial fall in systemic vascular resistance, which results in a blood pressure drop since the first trimester, and which is aimed to optimize the uteroplacental blood flow. This triggers profound adjustments in the whole cardiovascular system, mainly on myocardial contractility and heart rate, and renal hormonal activity, resulting in capillary engorgement and tissue edema, especially in the upper airway. In the trauma setting, this may increase even further the risk of failed intubation and complications related to hemodynamic instability (11).

An increased circulating blood volume, primarily due to sodium retention via renin-angiotensin-aldosterone system activation, and a consensual decrease in red blood cell count and hemoglobin concentration account for the relevant hemodilution that is pivotal to maintain blood pressure and placental perfusion (12). In a trauma pregnant patient, this relative anemia state may lead to a delay in hemorrhagic shock recognition with potential hazardous effects on the fetus, despite the apparent clinical stability of the mother (13).

Despite the platelet count progressively falls during gestation, the concentration of many clotting factors (e.g. VIII, IX, X, and fibrinogen) raises and generates a general prothrombotic state that guarantees a solid hemostasis response, preventing acute blood loss after the delivery (11). Hence, the pregnant patient has an increased risk for deep vein thrombosis (DVT) and disseminated intravascular coagulation (DIC) (14, 15).

Regarding the respiratory system, the expanding uterus causes diaphragmatic upward displacement with a consequent reduction in all lung volumes and physiological dyspnea. In order to provide oxygen to the fetus, both metabolic rate and maternal oxygen consumption increase. This, combined with the stimulant effect of progesterone, triggers maternal compensation resulting in augmented respiratory rate and tidal volume (16). Clinicians must keep in mind that increased oxygen consumption may lead to rapid episodes of desaturation, in particular during intubation. Providing supplemental oxygen to avoid maternal and fetal hypoxia, especially in a trauma setting, is compulsory (17).

**Figure 2:**
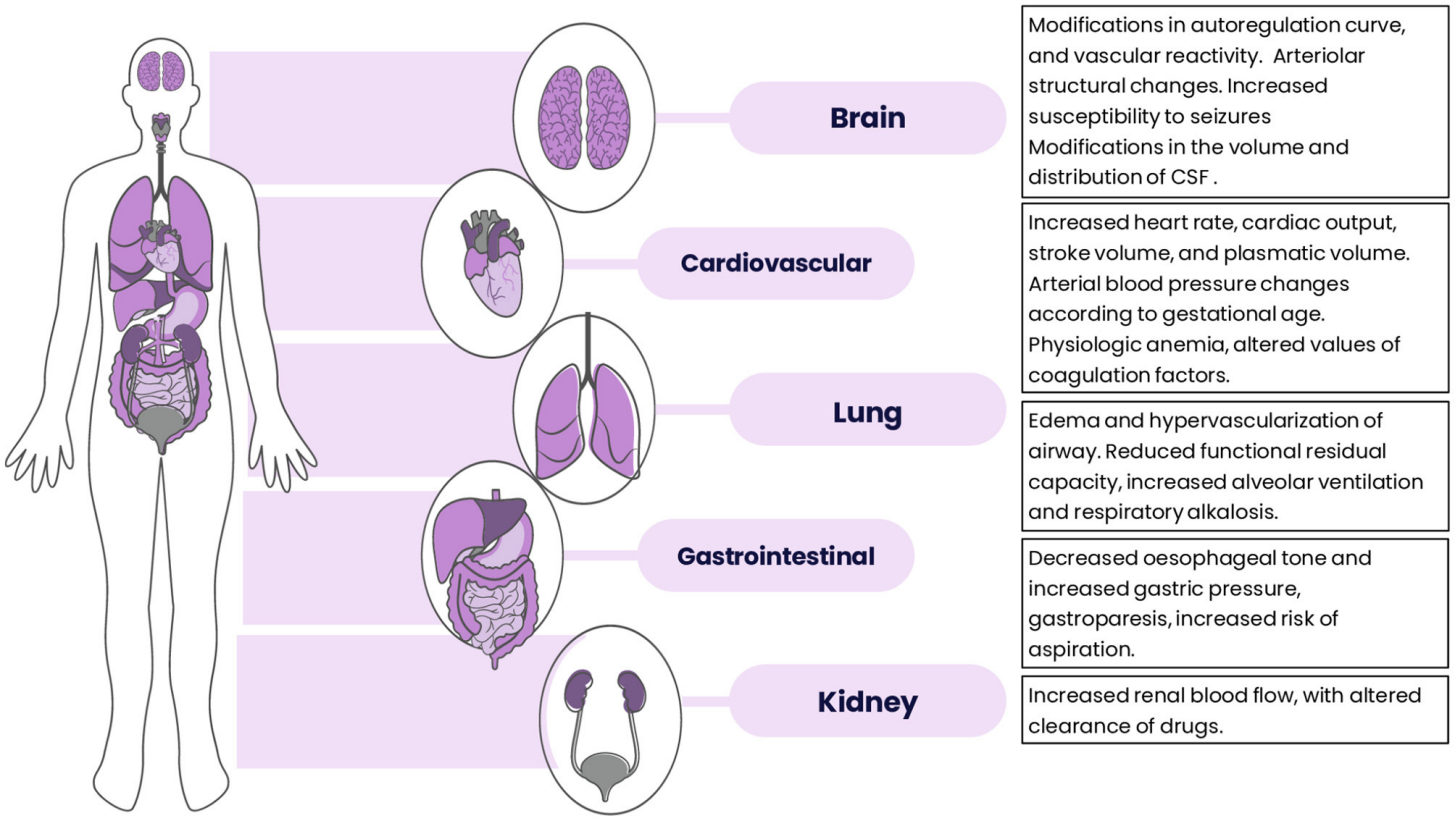
Pathophysiological changes that occur during pregnancy. CSF, cerebrospinal fluid.

To summarize, significant anatomic, biochemical and mechanical modifications occur during normal pregnancy with the purpose to sustain the growing fetus. A basic understanding of these changes could help physicians to discern between symptoms that mimic a disease and an actual condition requiring proper medical intervention. Other pathophysiological changes occurring during pregnancy are presented in Figure 2.

### Rule 2. You Have to Categorize the Mother and the Fetus Risks and Benefits

Physician should always suspect and exclude pregnancy in any female of childbearing age admitted for trauma (18).

During the first and second trimester the fetus is consensually considered non-viable (<23 week), thereby, timely maternal assessment and stabilization via Advanced Trauma Life Support (ATLS) protocols is the absolute priority. In fact, the stabilization of mother's condition in the early phases further optimizes fetal status. If the patient undergoes surgical intervention, the management of post-operative course and Intensive Care Unit (ICU) management should follow the general principles of the Brain Trauma Foundation Guidelines (5).

During the third trimester the fetus is considered viable (>23 weeks), unless proven otherwise. If the pregnant's clinical status is stable, the viability of the fetus should be carefully documented and monitored in order to establish the appropriate time and method of delivery (cesarean section alone or simultaneous with neurosurgery). Most of the neurosurgical trauma-related emergencies (e.g., hematoma, contusion, edema) demand the control of increased ICP (19). The aim of surgery is to relieve high ICP by mass evacuation, drainage of cerebrospinal fluid or decompressive craniotomy. This is primarily done to avoid further damage to neural structures and increase the chance of maternal survival improving the outcome of both mother and fetus (20, 21).

On the contrary, if maternal condition is critical or cerebral damage leads to maternal brain death, urgent cesarean delivery should be the priority.

In some cases, surgical treatment may lead to a favorable prognosis for both mother and fetus. If the fetus manifests signs of distress, despite an efficient maternal resuscitation, a cesarean delivery must be promptly performed. In the worst scenario, a fetal demise may be managed conservatively *in-utero* in the effort to firstly optimize maternal vital functions (8).

### Rule 3. Priorities in Initial Resuscitation

When a traumatic event occurs, an early targeted maternal resuscitation is the priority for the clinicians. Similarly to non-pregnant traumatic patient, primary survey after TBI should initially assess maternal vital functions. General principles based on the ABCDE mnemonic approach (Airway, Breathing, Circulation, Disability, Exposure) should be adopted to the specific needs of this population (22).

A: Pregnant patients have an augmented risk of failed intubation and acidic gastric contents aspiration compared to the non-pregnant population. Early placement of an endotracheal and a nasogastric tube is recommended to secure the airway. If signs of basilar skull fractures are found, the tube should be temporarily inserted through the mouth until a CT scan is performed (18).
B: Oxygen supplementation is mandatory to maintain a saturation > 95% in order to prevent both maternal and fetal hypoxia (23).
C: The target for initial resuscitation is a systolic blood pressure of 80–100 mmHg, but this may depend on patients autoregulatory status. Administration of crystalloids and blood products through a double intravenous line is paramount. Specific attention should be given to patient's position in order to prevent supine hypotension, as the fetus can cause venae cava collapse and reduction of venous return and cardiac output. Left-tilt or manual uterine displacement should be accomplished.
  The use of high dose norepinephrine should be avoided to preserve placental perfusion. The first-line vasopressor is phenylephrine, an α-1 agonist that does not modify uterine tone. 0-negative blood type should be transfused in order to avoid Rh sensitization (9, 24).
D: TBI mandates a quick but accurate neurological assessment of the patient. Glasgow Coma Scale (GCS) is the most widely used score to evaluate level of consciousness and to classify the severity of TBI, albeit with many limitations. More recently, the Full Outline of Unresponsiveness (FOUR) score, was introduced. Compared to GCS, this scale offer a more comprehensive assessment of patient's neurological status, including information on brainstem reflexes, breathing patterns and respiratory drive (25, 26).
E: Once the cardiopulmonary system has been stabilized, the body of the victim should be entirely exposed and carefully examined with special regard to thorax, abdomen, pelvic and perineal regions to rule out fetal injuries (18). Avoiding extreme hypothermia is mandatory (27).

In addition, further special consideration should be paid to acute spinal chord injury (SCI). The immediate resuscitation measures after SCI should include simultaneously maternal stabilization and fetal monitoring in an effort to timely counteract the detrimental consequences of the most fearsome occurrence, neurogenic shock (28, 29). Treatment of neurogenic shock includes hemodynamic stabilization by administering intravenous fluids to prevent secondary brain injury and fetal hypoxia. In case of refractory shock, vasopressors and inotropes should be considered to maintain an adequate organ perfusion pressure (30).

Initial resuscitation measures could not be considered accomplished without an obstetric evaluation of the fetus. In the trauma scenarios, acute brain injury alone can lead to fetal damage; therefore, it is not surprising that concomitant lesions in other body districts increase the risk of pre-term delivery or even fetal demise. Placental abruption and uterine rupture are the leading causes of fetal death (31). In this context, sonography may be a helpful adjunct to the primary survey, in both obstetrics and gynecology, providing immediate confirmation of findings obtained at bedside. The Focused Assessment with Sonography in Trauma (FAST) examination is a comprehensive study that could quickly detect free fluids in the abdomen and pelvis, fetal heart movements and placenta anomalies triggering proper medical or surgical intervention (32, 33).

Although the initial resuscitation maneuvers are identical for every patient, pregnant women require a special attention as even mild head injuries could put maternal and fetal life at risk (34). Clinicians should keep in mind that they are resuscitating two interrelated patients simultaneously and a multidisciplinary approach is often required for a successful outcome.

### Rule 4. Computed Tomography Scan Is the Neuroimaging of Choice to Evaluate TBI

When a TBI occurs, an exhaustive radiological study is mandatory in order to identify life-threatening injuries and evaluate the trauma extension. Unfortunately, radiodiagnostic procedures in pregnant women are frequently delayed by clinicians because of the fear to expose the developing fetus to harmful ionizing radiations (IR) (8). IR have a dose-dependent teratogenic and carcinogenic potential, especially when organogenesis occurs (5–10 weeks of gestation). Analogously, after 10 weeks, IR could produce delayed growth or injuries to the central nervous system. Nonetheless, most of the radiological studies, including head Computed Tomography (CT) scan, expose the fetus to a radiation amount below the threshold of 5,000 mrad, considered safe for fetal damage (Table 1) (18, 35).

**Table 1:** Estimated fetal radiation adsorbed doses during some common radiodiagnostic procedures (adapted from Ratnapalan et al.) (35).

**Examination**	**Fetal dose (mrad)**
**X-Ray**	
Upper gastrointestinal series	100
Cholecystography	100
Lumbar spine radiography	400
Pelvic radiography	200
Hip and femur radiography	300
Retrograde pyelography	600
Abdominal radiography	250
**Lumbar spine:**	
• Anteroposterior	750
• Lateral	91
• Oblique	100
Barium enema	1,000
Intravenous pyelogram	480
**Computed tomography**	
Head	0
Chest	16
Abdomen	3,000

*mrad, millirad*.

CT scan is a rapid and accurate procedure that appears to be the gold standard for the initial evaluation of TBI, as it can provide information on the need of surgical intervention and monitoring. Moreover, Gadolinium-based medium administration should be considered if maternal benefit outweighs fetal risks since there is no evidence of teratogenic effects in humans (18).

In conclusion, IR represent a possible cause of fetal injury and this should not be ignored. Nonetheless, sporadic exposure from radiodiagnostic procedures do not increase the hazard of congenital anomalies (18). For this reason, physicians should be encouraged in ordering head CT scan without delay as it is a fundamental tool for a quick diagnosis which can potentially improve patient's survival. When possible, minimizing fetal exposure to X-rays, by shielding woman's abdomen, may be beneficial (18).

### Rule 5. Determine Fetal Viability Though Fetal Monitoring Is Fundamental

Pregnant patients with severe TBI are at increased risk of fetal loss as a consequence of maternal injuries. For this reason, special attention should be paid to the fetus using a multidisciplinary approach including obstetrics, gynecologists and intensivists. The assessment of fetal well-being includes serial echographic approaches, electronic fetal heart rate monitoring (EFM), obstetric anamnesis and physical examination (33).

The calculation of the correct gestational age, performing an ultrasound scan, is crucial to evaluate fetal viability and to interpret tests. While during early gestational age (<13 weeks) the fetus is protected by the pelvic bones, and the cause of fetal loss is mainly placental hypoperfusion, in the late stages the unborn child is more subject to life-threatening trauma-related injuries. As aforementioned, a fetus is generally considered viable after 23 weeks of gestation. Before this age, there is a poor chance of survival outside the womb, especially after a major trauma (18).

In case of major trauma the priority falls toward mother's assessment and stabilization but, if the fetus is viable, EFM should be initiated as soon as possible and continued for at least 4 hours. This powerful tool provides information about fetal health status and, in case of abnormal heart rate pattern (early warning signal of maternal hemodynamic compromise and uteroplacental hypoperfusion), it allows to rapidly assess and prevent reversible causes such as hypoxia and acidosis. Noteworthy, most anesthetic drugs utterly decrease the physiological fetal heart rate variability (36), and this has to be kept in mind in the perioperative settings.

In summary, a major trauma mandates an obstetrical consultation, that should be promptly performed, in the effort to rule out uteroplacental involvement, maternal hemorrhage, fetal trauma-related injuries and viability.

### Rule 6. Avoid Secondary Insults of Systemic Origin

Management of TBI includes the treatment of the primary brain injury (hematoma, hemorrhage, seizures, cerebral edema), and the avoidance of secondary brain damage, and detrimental extra-cerebral secondary insults (37). Secondary brain damage, that is caused by the physiological responses to the initial lesion, is an injury whose effects do not occur at the time of the trauma but becomes evident in the following hours or days (38, 39). These insults, both ischemic and non-ischemic, are equally dangerous for brain health status and should be timely recognized and treated. Common post-traumatic complications include fever, hyperglycemia, anemia and hypernatremia. Unsurprisingly, these findings are strongly associated with poor outcome and increased mortality (40).

Recently, a new acronym was introduced, GHOST-CAP, that may be helpful to easily remind the key aspects involved in the etiology of secondary brain injury and to improve the quality of patient care (37). Although it was designed for any critical patient with acute brain injury, it can be extended to pregnant patients with TBI with no restrictions, and should be adapted to physiological changes occurring in pregnancy.

The GHOST-CAP mnemonic highlights eight pivotal elements that should be regularly assessed during patient's ICU stay.

#### Glycemia

Glucose plays a critical role in cerebral metabolism, but patients with acute brain injury may not require a strict glycemic control by insulin infusion as a result of an increased metabolic demand after the trauma. Hypoglycemia (<100 mg/dl) should be avoided (41).

Hemoglobin: anemia in critically ill patient is a multifactorial condition (e.g., blood loss, hemodilution). As an important variable of oxygen delivery (DO_2_) equation, a significant reduction of Hemoglobin (Hb) is associated with decreased cerebral DO_2_ and poor outcome after TBI. A reasonable Hb value should range between 7 and 9 g/dl (42, 43).

#### Oxygen

Hypoxemia is a well-known determinant of brain injury. Interestingly, different clinical studies suggest that supplemental oxygen administration toward hyperoxemic ranges is associated with worse outcome and increased mortality rates in TBI. Targeting SpO_2_ between 94 and 97% is recommended (44).

#### Sodium

Na^+^ is the main extracellular ion that regulates water movement across cell membranes, including neurons. Thereby, changes in sodium concentration can alter brain volume. Sodium levels outside the physiological limits are associated with poor outcomes in acute brain injury (45). Excessive correction of sodium plasma concentration may be harmful. Sodium levels up to 155 mEq/L may be tolerated (37).

#### Temperature

Hyperthermia has many etiologies, both septic and non-septic. An elevated temperature in TBI is associated with poor outcome as thermotoxicity involves cellular, local and systemic mechanisms. A core temperature >38°C (100 °F) should be avoided (46). Mild induced hypothermia may be considered in case of refractory intracranial hypertension, although it is not free from maternal and fetal side effects (47, 48).

#### Comfort

Avoiding patient physical distress (pain, restlessness, shivering) is essential to prevent harmful rises in ICP. Sedation and analgesia are valid therapeutic options to decrease ICP (49).

#### Arterial Pressure

CPP is the driving force that provides perfusion and oxygen to the brain and strictly depends on ICP and mean arterial pressure (MAP). Hence, abnormally low blood pressure can compromise cerebral perfusion and must be promptly corrected. Ultimately, the target MAP should be adjusted to maintain a CPP of at least 60 mmHg.

#### PaCO_2_

Changes in PaCO_2_ cause consensual variations in cerebral blood flow (CBF) and, ultimately, in ICP. Hypercapnia may result in intracranial hypertension (IHT). Similarly, a PaCO_2_ < 35 mmHg should be initially avoided in order to prevent cerebral ischemia (4).

In addition, TBI is a well-recognized cause of seizures and epilepsy, especially in pregnant women where a modification of seizure propensity can be observed physiologically (50). Unfortunately, to date, there are insufficient evidences regarding the management of post-traumatic seizures (PTS) during gestation.

PTS can occur early (within a week after trauma) as a direct consequence of the injuries or manifest later resulting more likely in post-traumatic epilepsy (PTE). Importantly, both early and late seizures can potentially exacerbate the secondary brain injury associated to trauma (51). Prophylactic therapies are not recommended, but in the suspect of diagnosis of seizures, the use of some antiepileptic drugs (AED), such as carbamazepine and valproate, can be considered as an option. However, the use of AED in this population may give rise to concerns about potential adverse drug reactions on the fetus, leading to major congenital malformations and other disabling anomalies (52). To mitigate the worries, recent studies conducted on epileptic pregnant women showed that the great majority of infants, exposed to AED *in-utero*, especially as part of a monotherapy regimen, did not develop any deformity (53, 54).

In conclusion, despite pharmacokinetic changes in pregnancy and licit fetal concerns, administering AED as a monotherapy to treat PTS and PTE may be appropriate and advantageous to improve both maternal and fetal outcomes (55).

### Rule 7. Specific Neuromonitoring: When? How? Invasive vs. Non-invasive

The gold standard for neuromonitoring in moderate to severe TBI is invasive ICP measurement obtained through a catheter insertion either into cerebral parenchyma or ventricles according to the clinical picture and center practice (56, 57). Several studies conducted in acute TBI adult patients observed that IHT represents a risk factor for mortality or poor neurological outcome (9). Moreover, combining invasive ICP with advanced pressure of brain tissue oxygen (PbtO_2_) monitoring may potentially improve patient outcome (58). Therefore, the indications for invasive neuromonitoring in pregnant patients should be the same as for the general ICU population (5).

Non-invasive techniques, such as transcranial doppler ultrasonography (TCD), electroencephalogram (EEG), near-infrared spectroscopy (NIRS) and optic nerve sheath diameter (ONSD), can be additional monitoring tools aimed to assess cerebrovascular dynamics and function. However, these methods are not accurate enough to substitute invasive techniques despite having the advantage of being safe and repeatable (59).

- TCD is a powerful harmless tool able to estimate some complications associated with TBI, such as hypoperfusion, hyperemia, vasospasm. Some formulas applied to calculate ICP and CPP values are available but they remain to be fully validated.
- EEG is a crucial diagnostic technique routinely employed in TBI patients, especially in the diagnosis of seizures and non-convulsive status.
- NIRS is a method able to assess neurologic impairment after TBI by the early detection of changes in regional cerebral oxygen saturation (rSO_2_). For this reason, it could be employed as an additional tool in the management of TBI and in those cases where invasive monitoring is proscribed.
- ONSD is an echographic exam that evaluates the optic nerve sheath expansion in presence of IHT. Although this method has several limitations, it could be an additional useful tool when invasive techniques are not feasible (59, 60).

### Rule 8. Approach to Intracranial Hypertension During Pregnancy

It is widely known that several physiological changes occur at many levels in the central nervous system during pregnancy. Although these adaptions act to preserve brain homeostasis, they can lead to neurological complications and IHT as a result of an exacerbated preexisting pathology or an unexpected acute event (9). Except for preeclampsia, head trauma is the most frequent cause of IHT during gestation (61).

IHT is a key actor in TBI since it closely correlates with CPP. Sustained IHT reduces CPP, resulting in cerebral ischemia and neurological deterioration (62). The maintenance of adequate CPP and ICP remains a primary target to improve prognosis in head injured pregnant patients (22).

Recent evidence suggests that the critical thresholds for CPP and ICP after a traumatic head injury are values of 60–70 mmHg and 22 mmHg respectively (63, 64). Nonetheless, a fixed threshold may not be appropriate for every patient since more vulnerable subgroups, as pregnant trauma patients, may require different strategies (5, 63). Moreover, physicians have to keep in mind that MAP -and therefore CPP- significantly changes during pregnancy with a gradual fall during early gestational age and a return to preconception levels thereafter (65).

**Figure 3:**
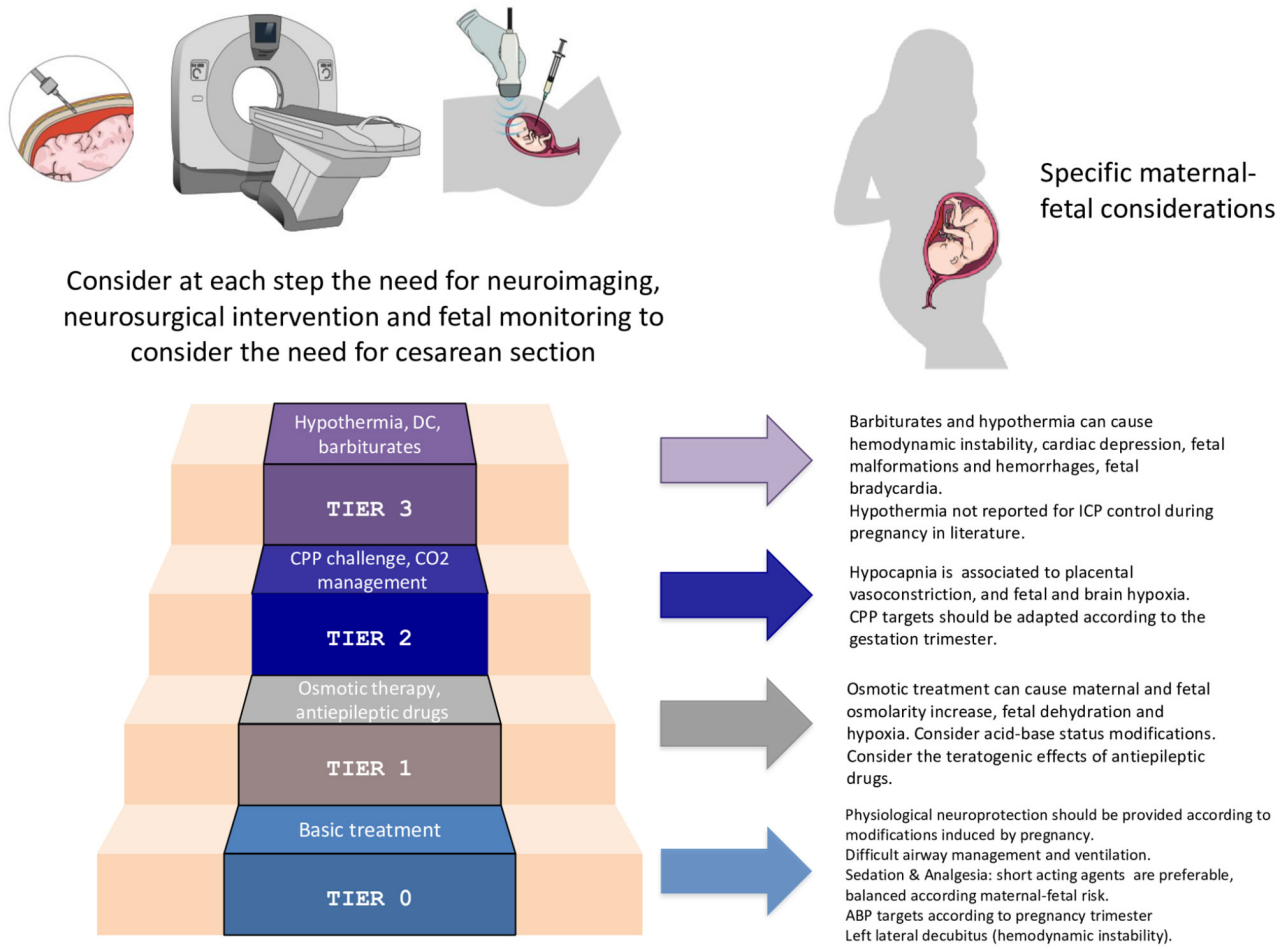
Specific considerations for the application of the staircase approach for intracranial pressure management in the pregnant woman. ICP, intracranial pressure; CPP, cerebral perfusion pressure; DC, decompressive craniotomy; ABP, arterial blood pressure.

As regards IHT management, according to the recently-published consensus-based protocol of Seattle, ICP treatment should follow a stepwise approach (64). Appropriate interventions range from general clinical management (safer for both mother and fetus) to more aggressive treatments (Figure 3).

Basic interventions, as head elevation, end-tidal CO_2_ monitoring, neurological assessment, physiological neuroprotection, in virtue of their few side effects, should be employed first.

Switching to higher tiers (e.g., AED, mannitol and vasopressor administration, mild hypocapnia, curarization) might be necessary for IHT control. Nonetheless, osmotic treatment can cause maternal-fetal hyperosmolarity and fetal dehydration. AED may have teratogenic effects, whereas hypocapnia can cause placental vasoconstriction and fetal hypoxemia.

If the patient is not responding to these treatments, more aggressive strategies by employing barbiturates, performing a decompressive craniectomy or using hypothermia can be taken in considerations. However, these interventions may have considerable negative consequences on fetal well-being, and mother and fetus should be frequently reassessed in order to rule out reversible or extra-cranial causes of treatment resistance.

Some treatments, that in first instance may seem beneficial (e.g., routinely use of furosemide or corticosteroids, continuous intravenous infusion of mannitol, extreme hyperventilation and hypothermia), are proscribed in the management of TBI (9, 64).

Regarding neurosurgery, literature on pregnant patients is limited and anesthesiologic management is mainly based on theoretical principles and case reports. Neuroanesthetic approach during pregnancy poses multiple challenges and should provide a balance between maternal favorable outcome and fetal well-being in a multidisciplinary way (66). Clinicians have to keep in mind that any perioperative procedure may predispose the patient to new-onset secondary brain injuries and may affect fetal status (67, 68).

In conclusion, IHT is a common finding after an acute head trauma during gestation but its management is far from being standardized, mainly because of lack of clinical evidence. To date, most of the literature extrapolates data from non-pregnant patient trials. Thereby, IHT management protocols in pregnancy should be wisely adjusted to increase maternal and fetal survival.

### Rule 9. When to Induce Labor? Natural or Caesarean Section?

TBI management during pregnancy can be challenging for healthcare providers due to the contemporary presence of two patients. Trauma has fetal repercussions and its effect on pregnancy depends mainly on the gestational age and on the injury severity. In this setting, a multidisciplinary team is compulsory. In particular, the trauma team must closely interact with the obstetrician whose role is to assess fetal status and to help in making decision about delivery weighting fetal benefits against maternal risks (33, 69).

In selected cases, preterm delivery may be the best choice, particularly when delivery may improve mother's prognosis. On the other hand, if definitive treatment can be safely delayed, and the gestational age is appropriate, additional stay in the ICU, to guarantee *in-utero* fetal development, possibly administering tocolytic agents (such as nifedipine or oxytocin antagonists), can be undoubtedly beneficial for the fetus (61). As mentioned above, after 23 weeks of gestation the fetus is considered viable and glucocorticoids should be administered to promote fetal lung maturation and to prevent fetal respiratory distress syndrome (70). According to a recent meta-analysis, after 32 weeks of gestation no further prolongation of pregnancy seems necessary (71).

Regarding the route of delivery, although in nearly all cases of TBI reported in literature more cesarean sections were performed, vaginal delivery may be accomplished (70, 72).

Specifically, cesarean section should be reserved to cases when maternal injuries result in severe complications such as fetal distress, placental abruption, uterine rupture or preterm labor, according to the gestational age (69). By contrast, in case of maternal stability, induced vaginal delivery should be considered in view of a non-viable fetus (8).

Perimortem cesarean section, defined as the surgical delivery of the fetus performed after maternal cardiac arrest, should start within 4 minutes after unsuccessful cardiopulmonary resuscitation for a maximum beneficial outcome. Interestingly, as the gravid uterus increases the oxygen demand and reduces venous return and stroke volume, in some cases, the emergency cesarean section may be life-saving for both mother and fetus (24).

A collegial decision-making should address the management of cesarean delivery, especially regarding the timing of the cesarean section which may be performed before or after neurosurgery. In the first case, general anesthesia is indicated for surgical delivery, even if some precautions on drugs choice, especially inhalation agents, should be taken into consideration. This strategy prevents the fetus from exposure to some treatments employed during neurosurgery, such as hyperventilation, mannitol, barbiturates and opioids and at the same time makes the mother more manageable (73, 74).

On the other hand, if uncomplicated neurosurgery is achieved, and the patient's ICP is within normal range, a neuraxial technique (epidural or spinal anesthesia) may be used in order to deliver the viable baby. However, despite the many undoubted advantages, the choice of this techniques is not risk-free as hypotension, dural puncture or epidural hematoma could have devastating consequences (67, 73).

### Rule 10. Ethical-Legal Aspects

Refractory IHT may result in maternal brain death (BD), a serious clinical condition also known as cerebral death or death by neurological criteria (DNC) (75). According to the most recent definition, BD, that was first described in 1959 (76), is the complete and permanent loss of brain function with loss of consciousness, brainstem reflexes and independent breathe (75).

In most instances, this severe condition results in maternal and fetal demise. However, thanks to the enormous advances in life support technologies and critical care management, to date, the progression of pregnancy is possible and a viable newborn can be delivered, despite the increased probability of complications (77). In some cases, interventions aimed to sustain patient's vital functions, after a BD diagnosis, are justified for the sake of the fetus, whose survival depends first on the gestational age. However, the dilemma whether or not to offer somatic care to a brain-dead pregnant patient has become a controversial ethical issue and matter of strong debate (78).

In 2011, a Committee for the Ethical Aspects of Human Reproduction and Women's Health stated six recommendations for the management of BD during pregnancy. They concluded that the wishes of the patient, in relation to the withdrawal of life support in state of pregnancy, if expressed in life, are paramount. The woman has the right to die in dignity, in accordance with the principle of autonomy. Hence, physicians are not exonerated from the duty to respect this right and any inconsistency must be discussed with decedent's family members or surrogates (79). On the other hand, in the absence of any expressed wish, ethical principles of beneficence and non-maleficence and justice prevail (72). Once maternal concerns are fulfilled, the best interests of the fetus should be also assessed. The principal issues include fetal viability and beneficial long-term prognosis. All necessary efforts should be made to prolong pregnancy and improve fetal maturity if maternal condition is stable. On the contrary, if severe complications or signs of distress are documented in maternal-fetal unit, supporting an *in-utero* fetal demise may be appropriate (79). Always respecting either patient or relatives' wishes, a brain-dead mother should be considered as a potential organ donor after the delivery (70). In any case, current local laws, that may vary in different regions, should be always considered (78).

The management of a DNC in a pregnant patient after a TBI requires a multidisciplinary approach which should follow the current recommendations available. Despite the prolongation of pregnancy in a brain-dead woman is within the reach of current medicine, it is always recommendable to refrain from excessive medical interventions that are no longer beneficial and could expose the mother and the fetus to dispensable suffering.

## Conclusions

TBI in pregnancy is one of the leading causes of maternal death for non-obstetric reasons and, whether moderate or severe, is associated with unfavorable maternal and fetal outcomes. The complex physiological modifications occurring in pregnancy have a crucial role in the management of TBI in these patients. In addition, fetal concerns, and paucity of high-quality evidence further complicate the issue. Estimating the gestational age has a central role in fetal assessment and allows to choose the optimum timing for potentially life-saving surgery. Maternal assessment and stabilization appear to be an absolute priority and, in some cases, clinicians must weight maternal risks and benefits in order to offer the proper treatment. Although some major progresses have been achieved, to date, TBI management during gestation remains empirical and extrapolated from data on non-pregnant patients. Hence, treatment protocols should be adjusted accordingly to optimize maternal and fetal outcomes.

## Author Contributions

All authors listed have made a substantial, direct, and intellectual contribution to the work and approved it for publication.

## Conflict of Interest

The authors declare that the research was conducted in the absence of any commercial or financial relationships that could be construed as a potential conflict of interest.

## Publisher's Note

All claims expressed in this article are solely those of the authors and do not necessarily represent those of their affiliated organizations, or those of the publisher, the editors and the reviewers. Any product that may be evaluated in this article, or claim that may be made by its manufacturer, is not guaranteed or endorsed by the publisher.

## References

[1] Capizzi A Woo J Verduzco-Gutierrez M. Traumatic brain injury. Med Clin North Am. (2020) 104:213–38. 10.1016/j.mcna.2019.11.001 32035565

[2] Shah AJ Kilcline BA. Trauma in pregnancy. Emerg Med Clin North Am. (2003) 21:615–29. 10.1016/S0733-8627(03)00038-5 12962349

[3] Moppett IK. Traumatic brain injury: assessment, resuscitation and early management. Br J Anaesth. (2007) 99:18–31. 10.1093/bja/aem128 17545555

[4] Hawryluk GWJ Aguilera S Buki A Bulger E Citerio G Cooper DJ. A management algorithm for patients with intracranial pressure monitoring: the Seattle International Severe Traumatic Brain Injury Consensus Conference (SIBICC). Intensive Care Med. (2019) 45:1783–94. 10.1007/s00134-019-05805-9 31659383 PMC6863785

[5] Carney N Totten AM O'Reilly C Ullman JS Hawryluk GWJ Bell MJ. Guidelines for the management of severe traumatic brain injury, fourth edition. Neurosurgery. (2017) 80:6–15. 10.1227/NEU.0000000000001432 27654000

[6] Leach MR Zammit CG. Traumatic brain injury in pregnancy. Handb Clin Neurol. Elsevier (2020). p. 51–61. 10.1016/B978-0-444-64240-0.00003-9 32768094

[7] Agrawal D Sharma B Dawar P Kalra A. Decompressive craniectomy in term pregnancy with combined cesarean section for traumatic brain injury. Neurol India. (2013) 61:423. 10.4103/0028-3886.117588 24005739

[8] Kho GS Abdullah JM. Management of severe traumatic brain injury in pregnancy: a body with two lives. Malays J Med Sci. (2018) 25:151–7. 10.21315/mjms2018.25.5.14 30914871 PMC6419882

[9] Godoy DA Robba C Paiva WS Rabinstein AA. Acute intracranial hypertension during pregnancy: special considerations and management adjustments. Neurocrit Care. (2022) 36:302–16. 10.1007/s12028-021-01333-x 34494211 PMC8423073

[10] Soma-Pillay P Nelson-Piercy C Tolppanen H Mebazaa A. Physiological changes in pregnancy. Cardiovasc J Afr. (2016) 27:89–94. 10.5830/CVJA-2016-021 27213856 PMC4928162

[11] Carlin A Alfirevic Z. Physiological changes of pregnancy and monitoring. Best Pract Res Clin Obstet Gynaecol. (2008) 22:801–23. 10.1016/j.bpobgyn.2008.06.005 18760680

[12] Pritchard JA Rowland RC. Blood volume changes in pregnancy and the puerperiumiii, whole body and large vessel hematocrits in pregnant and nonpregnant women. Am J Obstet Gynecol. (1964) 88:391–5. 10.1016/0002-9378(64)90440-5 14123412

[13] Petrone P Asensio JA. Trauma in pregnancy: assessment and treatment. Scand J Surg. (2006) 95:4–10. 10.1177/145749690609500102 16579248

[14] Gando S Wada H Thachil J. The Scientific and Standardization Committee on DIC of the International Society on Thrombosis and Haemostasis (ISTH). Differentiating disseminated intravascular coagulation (DIC) with the fibrinolytic phenotype from coagulopathy of trauma and acute coagulopathy of trauma-shock (COT/ACOTS). J Thromb Haemost. (2013) 11:826–35. 10.1111/jth.12190 23522358

[15] Lippi G Cervellin G. Disseminated intravascular coagulation in trauma injuries. Semin Thromb Hemost. (2010) 36:378–87. 10.1055/s-0030-1254047 20614390

[16] Hegewald MJ Crapo RO. Respiratory physiology in pregnancy. Clin Chest Med. (2011) 32:1–13. 10.1016/j.ccm.2010.11.001 21277444

[17] Penning D. Trauma in pregnancy. Can J Anesth. (2001) 48:R34–40. 10.1007/BF03028176 27688134

[18] Jain V Chari R Maslovitz S Farine D Maternal Fetal Medicine Committee Bujold E. Guidelines for the management of a pregnant trauma patient. J Obstet Gynaecol Can. (2015) 37:553–74. 10.1016/S1701-2163(15)30232-2 26334607

[19] Pervez M Kitagawa RS Chang TR. Definition of traumatic brain injury, neurosurgery, trauma orthopedics, neuroimaging, psychology, and psychiatry in mild traumatic brain injury. Neuroimaging Clin N Am. (2018) 28:1–13. 10.1016/j.nic.2017.09.010 29157846

[20] Stocchetti N Maas AIR. Traumatic intracranial hypertension. N Engl J Med. (2014) 370:2121–30. 10.1056/NEJMra1208708 24869722

[21] Subramanian R Sardar A Mohanaselvi S Khanna P Baidya D. Neurosurgery and pregnancy. J Neuroanaesthesiol Crit Care. (2014) 01:166–72. 10.4103/2348-0548.139095

[22] Dinsmore J. Traumatic brain injury: an evidence-based review of management. Contin Edu Anaesth Crit Care Pain. (2013) 13:189–95. 10.1093/bjaceaccp/mkt01028745856

[23] Darlan D Prasetya G Ismail A Pradana A Fauza J Dariansyah A. Algorithm of traumatic brain injury in pregnancy (perspective on neurosurgery). Asian J Neurosurg. (2021) 16:249. 10.4103/ajns.AJNS_243_20 34268147 PMC8244712

[24] Mendez-Figueroa H Dahlke JD Vrees RA Rouse DJ. Trauma in pregnancy: an updated systematic review. Am J Obstet Gynecol. (2013) 209:1–10. 10.1016/j.ajog.2013.01.021 23333541

[25] Wijdicks EFM Bamlet WR Maramattom BV Manno EM McClelland RL. Validation of a new coma scale: the four score. Ann Neurol. (2005) 58:585–93. 10.1002/ana.20611 16178024

[26] Sadaka F Patel D Lakshmanan R. The four score predicts outcome in patients after traumatic brain injury. Neurocrit Care. (2012) 16:95–101. 10.1007/s12028-011-9617-5 21845490

[27] Aggarwal R Soni K Trikha A. Initial management of a pregnant woman with trauma. J Obstet Anaesth Crit Care. (2018) 8:66. 10.4103/joacc.JOACC_4_18

[28] Dawood R Altanis E Ribes-Pastor P Ashworth F. Pregnancy and spinal cord injury. Obstet Gynecol. (2014) 16:99–107. 10.1111/tog.12083

[29] Popov I Ngambu F Mantel G Rout C Moodley J. Acute spinal cord injury in pregnancy: an illustrative case and literature review. J Obstet Gynaecol. (2003) 23:596–8. 10.1080/01443610310001604321 14617457

[30] Dave S Cho JJ. Neurogenic Shock. Treasure Island: Stat Pearls Publishing (2022).

[31] Battaloglu E McDonnell D Chu J Lecky F Porter SK. Epidemiology and outcomes of pregnancy and obstetric complications in trauma in the United Kingdom. Injury. (2016) 47:184–7. 10.1016/j.injury.2015.08.026 26404664

[32] Richards JR McGahan JP. Focused assessment with sonography in trauma (FAST) in 2017: what radiologists can learn. Radiology. (2017) 283:30–48. 10.1148/radiol.2017160107 28318439

[33] MacArthur B Foley M Gray K Sisley A. Trauma in pregnancy: a comprehensive approach to the mother and fetus. Am J Obstet Gynecol. (2019) 220:465–8.e1. 10.1016/j.ajog.2019.01.209 30685288

[34] Fauzi AA. Pregnancy in head injury: friend or foe? A proposed guideline for the management of head injury in pregnancy. J Med Sci Clin Res. (2014) 2:18. Available online at: http://jmscr.igmpublication.org/v2-i4/30%20jmscr.pdf

[35] Ratnapalan S Bona N Koren G. Motherisk team ionizing radiation during pregnancy. Can Fam Physician. (2003) 49:873–4. Available online at: https://www.cfp.ca/content/cfp/49/7/873.full.pdf 12901480 PMC2214256

[36] Fedorkow DM Stewart TJ Parboosingh J. Fetal heart rate changes associated with general anesthesia. Am J Perinatol. (1989) 6:287–8. 10.1055/s-2007-999594 2730732

[37] Taccone FS De Oliveira Manoel AL Robba C Vincent J-L. Use a “GHOST-CAP” in acute brain injury. Crit Care. (2020) 24:89. 10.1186/s13054-020-2825-7 32171298 PMC7071769

[38] Siesjo BK Siesjo P. Mechanisms of secondary brain injury. Eur J Anaesthesiol. (1996) 13:247–68. 10.1046/j.1365-2346.1996.00976.x 8737117

[39] Huffman JC Brennan MM Smith FA Stern TA. (2010). Patients with Neurologic Conditions I. Seizure Disorders (Including Nonepileptic Seizures), Cerebrovascular Disease, and Traumatic Brain Injury. Massachusetts General Hospital Handbook of General Hospital Psychiatry. 237–53. 10.1016/b978-1-4377-1927-7.00019-4

[40] Wartenberg KE Schmidt JM Claassen J Temes RE Frontera JA Ostapkovich N. Impact of medical complications on outcome after subarachnoid hemorrhage. Crit Care Med. (2006) 34:617–23. 10.1097/01.CCM.0000201903.46435.35 16521258

[41] Hermanides J Plummer MP Finnis M Deane AM Coles JP Menon DK. Glycaemic control targets after traumatic brain injury: a systematic review and meta-analysis. Crit Care. (2018) 22:11. 10.1186/s13054-017-1883-y 29351760 PMC5775599

[42] Lelubre C Bouzat P Crippa IA Taccone FS. Anemia management after acute brain injury. Crit Care. (2016) 20:152. 10.1186/s13054-016-1321-6 27311626 PMC4911680

[43] Sekhon MS McLean N Henderson WR Chittock DR Griesdale DE. Association of hemoglobin concentration and mortality in critically ill patients with severe traumatic brain injury. Crit Care. (2012) 16:R128. 10.1186/cc11431 22817913 PMC3580711

[44] Vincent J-L Taccone FS He X. Harmful effects of hyperoxia in postcardiac arrest, sepsis, traumatic brain injury, or stroke: the importance of individualized oxygen therapy in critically ill patients. Can Respir J. (2017) 2017:1–7. 10.1155/2017/2834956 28246487 PMC5299175

[45] Sterns RH. Disorders of plasma sodium—causes, consequences, and correction. N Engl J Med. (2015) 372:55–65. 10.1056/NEJMra1404489 25551526

[46] Walter EJ Carraretto M. The neurological and cognitive consequences of hyperthermia. Crit Care. (2016) 20:199. 10.1186/s13054-016-1376-4 27411704 PMC4944502

[47] Urbano LA Oddo M. Therapeutic hypothermia for traumatic brain injury. Curr Neurol Neurosci Rep. (2012) 12:580–91. 10.1007/s11910-012-0304-5 22836524

[48] Hui J Feng J Tu Y Zhang W Zhong C Liu M. Safety and efficacy of long-term mild hypothermia for severe traumatic brain injury with refractory intracranial hypertension (LTH-1): a multicenter randomized controlled trial. EClinicalMedicine. (2021) 32:100732. 10.1016/j.eclinm.2021.100732 33681741 PMC7910713

[49] Oddo M Crippa IA Mehta S Menon D Payen J-F Taccone FS. Optimizing sedation in patients with acute brain injury. Crit Care. (2016) 20:128. 10.1186/s13054-016-1294-5 27145814 PMC4857238

[50] Chang BS Lowenstein DH. (2003). Practice parameter: Antiepileptic drug prophylaxis in severe traumatic brain injury: Report of the Quality Standards Subcommittee of the American Academy of Neurology. Neurology. 60:10–16. 10.1212/01.wnl.0000031432.05543.14 12525711

[51] Chartrain AG Yaeger K Feng R Themistocleous MS Dangayach NS Margetis K. Antiepileptics for post-traumatic seizure prophylaxis after traumatic brain injury. CPD. (2018) 23:6428–41. 10.2174/1381612823666171031100139 29086674

[52] Hernandez-Diaz S Mittendorf R Holmes L. Comparative safety of topiramate during pregnancy. Birth Defect Res A Clin Mol Teratol. (2010) 123:21–8. 10.1097/AOG.0000000000000018 24463659

[53] Morrow J. Malformation risks of antiepileptic drugs in pregnancy: a prospective study from the UK epilepsy and pregnancy register. J Neurol Neurosurg Psychiatry. (2006) 77:193–8. 10.1136/jnnp.2005.074203 16157661 PMC2077578

[54] Pennell PB. Antiepileptic drugs during pregnancy: what is known and which AEDs seem to be safest? Epilepsia. (2008) 49:43–55. 10.1111/j.1528-1167.2008.01926.x 19087117 PMC3882069

[55] Wlodarczyk BJ Palacios AM George TM Finnell RH. Antiepileptic drugs and pregnancy outcomes. Am J Med Genet A. (2012) 158A:2071–90. 10.1002/ajmg.a.35438 22711424 PMC3402584

[56] Berlin T Murray-Krezan C Yonas H. Comparison of parenchymal and ventricular intracranial pressure readings utilizing a novel multi-parameter intracranial access system. Springerplus. (2015) 4:10. 10.1186/2193-1801-4-10 25674495 PMC4320187

[57] Fernando SM Tran A Cheng W Rochwerg B Taljaard M Kyeremanteng K. Diagnosis of elevated intracranial pressure in critically ill adults: systematic review and meta-analysis. BMJ. (2019) 366:l4225. 10.1136/bmj.l4225 31340932 PMC6651068

[58] Hirschi R Hawryluk GWJ Nielson JL Huie JR Zimmermann LL Saigal R. Analysis of high-frequency PbtO2 measures in traumatic brain injury: insights into the treatment threshold. J Neurosurg. (2019) 131:1216–26. 10.3171/2018.4.JNS172604 30497191 PMC8979548

[59] Vinciguerra L Bösel J. Noninvasive neuromonitoring: current utility in subarachnoid hemorrhage, traumatic brain injury, and stroke. Neurocrit Care. (2017) 27:122–40. 10.1007/s12028-016-0361-8 28004334

[60] Khan M Shallwani H Khan M Shamim M. Noninvasive monitoring intracranial pressure – a review of available modalities. Surg Neurol Int. (2017) 8:51. 10.4103/sni.sni_403_16 28480113 PMC5402331

[61] Malaiyandi D James E Peglar L Karim N Henkel N Guilliams K. Neurocritical care of the pregnant patient. Curr Treat Options Neurol. (2021) 23:22. 10.1007/s11940-021-00676-2 34177249 PMC8214980

[62] Marmarou A Saad A Aygok G Rigsbee M. Contribution of raised ICP and hypotension to CPP reduction in severe brain injury: correlation to outcome. In: Poon WS Chan MTV Goh KYC Lam JMK Ng SCP Marmarou A Avezaat CJJ Pickard JD Czosnyka M Hutchinson PJA editors. Intracranial Pressure and Brain Monitoring XII. Acta Neurochirurgica Supplementum. Vienna: Springer Vienna (2005). p. 277–80 10.1007/3-211-32318-x_57 16463865

[63] Sorrentino E Diedler J Kasprowicz M Budohoski KP Haubrich C Smielewski P. Critical thresholds for cerebrovascular reactivity after traumatic brain injury. Neurocrit Care. (2012) 16:258–66. 10.1007/s12028-011-9630-8 21964774

[64] Chesnut R Aguilera S Buki A Bulger E Citerio G Cooper DJ. A management algorithm for adult patients with both brain oxygen and intracranial pressure monitoring: the Seattle International Severe Traumatic Brain Injury Consensus Conference (SIBICC). Intensive Care Med. (2020) 46:919–29. 10.1007/s00134-019-05900-x 31965267 PMC7210240

[65] Shen M Tan H Zhou S Smith GN Walker MC Wen SW. Trajectory of blood pressure change during pregnancy and the role of pre-gravid blood pressure: a functional data analysis approach. Sci Rep. (2017) 7:6227. 10.1038/s41598-017-06606-0 28740155 PMC5524922

[66] Kazemi P Villar G Flexman AM. Anesthetic management of neurosurgical procedures during pregnancy: a case series. J Neurosurg Anesthesiol. (2014) 26:234–40. 10.1097/ANA.0000000000000029 24296540

[67] Wang LP Paech MJ. Neuroanesthesia for the pregnant woman. Anesth Analg. (2008) 107:193–200. 10.1213/ane.0b013e31816c8888 18635488

[68] Sharma D Curry P Viernes D. Perioperative management of traumatic brain injury. Int J Crit Illn Inj Sci. (2011) 1:27. 10.4103/2229-5151.79279 22096771 PMC3209993

[69] Sim KB. Maternal persistent vegetative state with successful fetal outcome. J Korean Med Sci. (2001) 16:669. 10.3346/jkms.2001.16.5.669 11641542 PMC3057580

[70] Esmaeilzadeh M Dictus C Kayvanpour E Sedaghat-Hamedani F Eichbaum M Hofer S. One life ends, another begins: management of a brain-dead pregnant mother-a systematic review-. BMC Med. (2010) 8:74. 10.1186/1741-7015-8-74 21087498 PMC3002294

[71] Haas DM Imperiale TF Kirkpatrick PR Klein RW Zollinger TW Golichowski AM. Tocolytic therapy: a meta-analysis and decision analysis. Obstet Gynecol. (2009) 113:585–94. 10.1097/AOG.0b013e318199924a 19300321

[72] Siwatch S Rohilla M Singh A Ahuja C Jain K Jain V. Pregnancy in a persistent vegetative state: a management dilemma. Case report, literature review and ethical concerns. J Obstet Gynecol India. (2020) 70:310–3. 10.1007/s13224-019-01274-8 32764853 PMC7381523

[73] Heesen M Klimek M. Nonobstetric anesthesia during pregnancy. Curr Opin Anaesthesiol. (2016) 29:297–303. 10.1097/ACO.0000000000000311 26859466

[74] Bernstein K Hussey H Hussey P Gordo K Landau R. Neuro-anesthesiology in pregnancy. In: Handbook of Clinical Neurology. Amsterdam: Elsevier (2020). p. 193–204. 10.1016/B978-0-444-64239-4.00010-2 32736750

[75] Greer DM Shemie SD Lewis A Torrance S Varelas P Goldenberg FD. Determination of brain death/death by neurologic criteria: the world brain death project. JAMA. (2020) 324:1078. 10.1001/jama.2020.11586 32761206

[76] Mollaret P Goulon M. [The depassed coma (preliminary memoir)]. Rev Neurol. (1959) 101:3–15. 14423403

[77] Santos LA dos Pereira CU Paula MCG de Kalkmann GF Rabelo NN. Traumatic brain injury in pregnancy. Arquivos Brasileiros de Neurocirurgia: Brazilian Neurosurg. (2022) s-0041-1733862. 10.1055/s-0041-1733862

[78] Smok D Prager KM. The ethics of neurologically complicated pregnancies. In: Handbook of Clinical Neurology. Amsterdam: Elsevier (2020). p. 227–42. 10.1016/B978-0-444-64239-4.00013-8 32736753

[79] Dickens B. Brain death and pregnancy: FIGO Committee for the Ethical Aspects of Human Reproduction and Women's Health. Int J Gynecol Obstet. (2011) 115:84–5. 10.1016/j.ijgo.2011.07.002 21839449

